# En Caul Cesarean Delivery—A Safer Way to Deliver a Premature Newborn? Narrative Review

**DOI:** 10.3390/jcm14010051

**Published:** 2024-12-26

**Authors:** Izabela Pabin, Katarzyna Stefańska, Joanna Maria Jassem-Bobowicz, Dariusz Wydra

**Affiliations:** 1 Department of Gynecology, Obstetrics and Neonatology, Division of Gynecology and Obstetrics, Medical University of Gdańsk, 80-210 Gdańsk, Poland; 2 Department of Gynecology, Obstetrics and Neonatology, Division of Neonatology, Medical University of Gdańsk, 80-210 Gdańsk, Poland

**Keywords:** en caul, en caul delivery, en caul cesarean section, premature delivery

## Abstract

Premature deliveries and preterm newborns are of a special significance to obstetricians. Despite great improvement in neonatal intensive care in the last two decades, prematurity is still the leading cause of neonatal mortality and morbidity. Complications associated with premature deliveries are malpresentation, prolapse of the umbilical cord, entrapment of some parts of the fetal body, as well as severe bruising or bone fractures. The injuries may also include soft tissue damage, neurological injury, or intracranial hemorrhage. Small body weight as well as the unaccomplished development of fetal vital systems make preterm newborns vulnerable to delivery trauma. The main goal of a cesarean section in extremely preterm deliveries is to reduce the number of these complications. On the other hand, premature deliveries are associated with an undeveloped lower uterine segment and other difficulties encountered during the operation, which make the procedure more complicated and difficult to perform. Therefore, the preterm delivery or delivery of a fetus with growth retardation is of great concern. In our review, we investigated previous publications regarding en caul deliveries, mostly cesarean sections. We concentrated on the neonatal outcomes and tried to establish the optimal mode and time for a premature delivery.

## 1. Introduction

Premature deliveries and preterm newborns are of a special significance to obstetricians. Although the intensive care of preterm newborns is constantly being improved, preterm birth is still the leading cause of neonatal mortality and morbidity [1].

Complications associated with premature deliveries are as follows: malpresentation (including all types of breech presentation, which is most common in the early stages of pregnancy), prolapse of the umbilical cord (resulting from a disproportion in the circumferences of the fetal head and buttock), entrapment of some parts of the fetal body (due to contraction of the uterus), as well as severe bruising over the presenting part or bone fractures [1]. Small body weight as well as the unaccomplished development of fetal vital systems make preterm newborns vulnerable to delivery trauma. The main goal of cesarean sections was to reduce these possible complications. On the other hand, premature deliveries are associated with an undeveloped lower uterine segment and other difficulties encountered during the operation, which hinder the whole operation. Performing a cesarean section before 26 weeks of pregnancy is related to higher mother morbidity and mortality [2].

**Figure 1:**
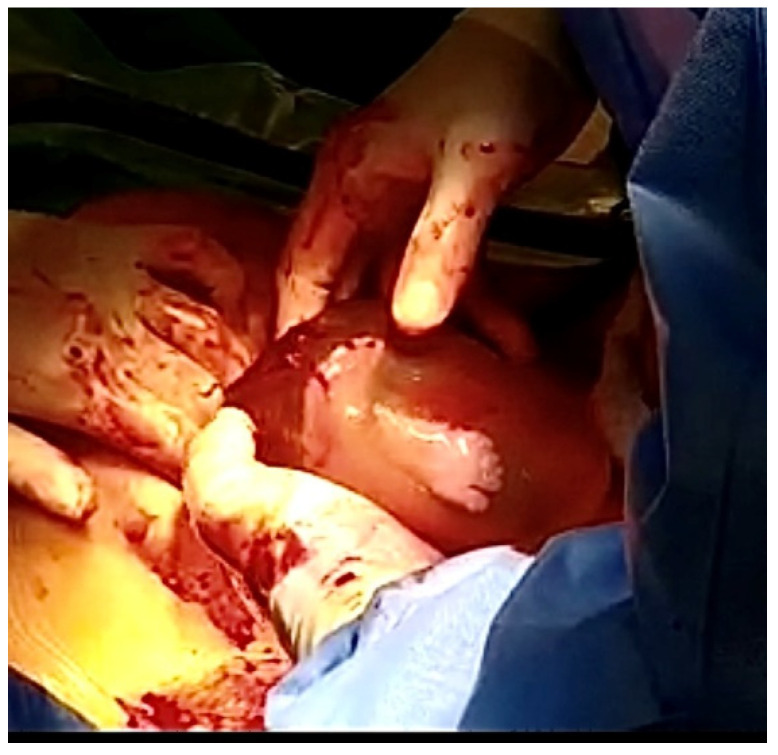
En caul delivery is a way of delivering a baby surrounded by the amniotic membranes and the amniotic fluid. (The picture was taken in the Department of Gynecology, Obstetrics and Neonatology, University Clinical Center in Gdańsk.)

The term “en caul delivery”, also known as a “veiled birth” or a “mermaid birth”, refers to a way of delivering a baby surrounded by the amniotic membranes and the amniotic fluid (Figure 1) [3]. We can also encounter the term “a caul birth” which means the delivery of a newborn with a part of the amniotic membrane attached to their body [4]. Deliveries “en caul”, or in the amniotic sac, may occur both in vaginal delivery or cesarean section. In the case of an “en caul” cesarean delivery, the term “amnion protective cesarean section” may be applied [5]. The incidence of such deliveries is reported to be from 1 in 80,000 to 1–2% [3,4,6]. This above all affects immature deliveries (born at the gestational age less or equal to 32 weeks) or cases of very low birth weight, when a child’s birth mass is estimated to be below 1500 g [1,4,5] or 1000 g [5,7].

As reported by National Vital Statistics reports from 2023, the preterm birth rate in the US rose in 2021 to 10.49% [8]. Other data state that about 30–35% of them are indicated, 40–45% are spontaneous, and 25–30% are evoked by the premature rupture of membranes [9]. Therefore, the need to search for optimal methods of delivery is crucial.

The aim of this study is an attempt to find a feasible way to improve neonatal outcomes in premature deliveries. We wanted to investigate the operation mode that seems to be less harmful and traumatic for both a newborn and the mother, bearing in mind possible complications and disadvantages.

## 2. Historical Meaning

Since ancient times, there have been strong magic beliefs associated with the caul. Its significance was mainly very positive. The membrane was supposed to bring good luck, fame, fortune, or love to its owner. It was carried as a charm and protection against sorcerers, witchcraft, and evil spirits. The powdered membrane was an ingredient of love potions or a remedy for malaria. The caul was also an object of great interest when it came to foretelling the future of a newborn, based on its color, consistency, and shape. On the other hand, it was associated with evil forces, clairvoyance, and the ability to communicate with ghosts. It was a prophecy either of death or becoming a witch, a sorcerer, or even a vampire [10]. It was even described in detail in an epic novel by Charles Dickens David Copperfield.

In the literature, we can encounter a first description of “en caul” delivery in 1975. In that case, a newborn lived for 25 min in the amniotic sac after the delivery and no negative consequences were reported at the age of 3 years old. It proves that placenta is able to maintain some oxygen supply enabling the newborn to survive for some time. Other pioneer publications are dated 1983 and 1984 [1].

## 3. Threats to Premature Newborns

The main goal of obstetricians worldwide is to ensure a safe delivery both for a child and the mother. A great concern is the preterm delivery or delivery of a fetus with growth retardation. Preterm birth is a major risk factor for neonatal mortality and morbidity [1]. It is known that the amniotic fluid and membranes serve as hydraulic protection to the fragile body of a newborn against the trauma of a delivery [11]. Premature newborns, especially extremely preterm ones, are vulnerable to birth trauma. Preterm, immature fetuses have an incompletely developed nervous system; their tolerance to hypoxia and mechanical stimulation is impaired and can be easily harmed during a delivery [5,8,12]. Injuries may include soft tissue damage, neurological injury, or intracranial hemorrhage, as well as causing bone fractures [1,6,7]. The damage may result from the pressure exerted on the fetus by a thick uterine wall or the hands of the operator [7]. The work by Blagaić et al. reported no bone fractures and showed a better physical appearance in the group delivered by “en caul” cesarean section [5]. The second technical difficulty is caused by a thick wall of the uterus and an underdeveloped lower segment making it more difficult to incise properly. A complication called “hug-me-tight-uterus” is a situation when the contracted walls of the uterus trap a fetus after a sudden break of waters during a delivery [1,7]. Another complication correlated with strong uterine contractions is a change of fetal presentation into a transverse lie after the rupture of membranes [1].

Intrauterine content decreases after the rupture of the amniotic sac which may increase the strength of uterine muscle contractions. This may lead to the compression of the umbilical cord and the aspiration of amniotic fluid by the fetus [13]. It is reported that extremely premature infants are prone to birth trauma after the rupturing of membranes, especially when the fetus is malpresented and the uterus contractions are strong [1].

The combination of those aspects resulted in attempts to deliver premature newborns within the amniotic sacs, which play a protective role and minimize the trauma of delivery. It is reported to be less traumatic [1,6]. Neonates with extremely low birth weight shall benefit most from this method of delivery [14].

## 4. Surgical Technique

The authors agree that establishing an optimal method of uterine incision is one of the most essential steps during the operation that can enhance the survival rate of newborns [1,5,7]. Murakoshi et al. provide a detailed description of the most optimal surgical technique to perform an “en caul” cesarean delivery [7].

Bearing in mind the possible harm done to the premature fetus and the technical difficulties of operating at such a low gestational age, Murakoshi proposes the mode of a U- or J-shaped incision of the lower segment, as a safe option enabling further wound extension, if necessary. However, such a need is reported very rarely [7].

To enhance the chances of a successful delivery, a spinal and/or epidural anesthesia is performed together with an intravenous application of an initial dose of 100 µg of nitroglycerin that may provide an appropriate relaxation of the uterine muscle. The dose may be repeated until adequate relaxation is obtained. Usually, 200–500 µg of nitroglycerin is required. It is called rapid tocolysis, as the result is observed within 60 s of administration and lasts for 5 min. Nitroglycerin may decrease blood pressure, and it is advised to administer an infusion of crystalloid or colloid fluid before the nitroglycerin. Also, ephedrine can be used if necessary [7].

Secondly, a laparotomy is performed. A small skin incision must be avoided. The authors underline the importance of ensuring a large enough uterine incision to enable the delivery of a baby within an intact amniotic sac [7].

The next step is a transverse U- or J-shaped incision of the lower segment of the uterus. It is very difficult as the isthmus is poorly formed and thick in the case of premature deliveries. Those types of incisions are easy to extend if necessary to provide an adequate entrance to the uterus. It is advisable to make a first U-shaped incision wide and shallow and follow by a deeper and narrower one until the membranes become visible. With the support of the middle and index fingers, extend the incision towards the round ligament to avoid lateral lacerations. All efforts must be taken to preserve the amniotic membranes and avoid the tearing of the uterine arteries and veins [5,6].

T-, J-, or U-shaped incisions of the uterus are variations in low transverse uterine muscle incisions. The low transverse incision may be extended to J- or inverted T-shaped incisions up to the level of the round ligament insertion in the case of a need of a large incision, mostly in the case of very premature deliveries [15,16,17,18]. On the other hand, there is a classical, vertical incision of the uterine contractile body.

No difference has been stated between vertical or transverse uterine incision in the incision-to-delivery time or short-term maternal and neonatal outcomes [17,19].

A classical or T-shaped incision of the uterus is correlated with a higher risk of uterine rupture in the case of subsequent pregnancies [1]. The vertical hysterotomy incision (classical cesarean section or T-shaped extension) is associated with an increased risk of maternal morbidity such as infection, postpartum hemorrhage, transfusion, and maternal intensive care unit admission compared to a low transverse incision [18,19,20]. Advantages of the transverse incision of the uterus are associated with less blood loss, less need for bladder dissection, the ease of repairing the uterus wound, as well as minimized adherence of the omentum or bowels to the site of the incision [1,5,16,17,21].

Moreover, a lower incidence of uterine rupture in subsequent pregnancies makes the lower uterine incision the best option in patients planning another pregnancy and allows an attempt of vaginal labor in the following pregnancy and therefore may help to avoid future cesarean sections [15,17,22].

U-shaped myometrium incision prevents injury to the vascular plexus of the uterine lateral wall and can also avoid myoma of the uterus and thick vessels [23]. The risk of injuring large branches of the uterine artery resulting in increased maternal blood loss is still present in the case of a J-shaped incision [17].

In the case of premature cesarean delivery, the undeveloped lower uterine segment wall is thicker and has a larger surface area of the transected myometrium that may lead to larger blood loss with the lower uterine incision. Kawakita proved no difference in intraoperative complications such as estimated blood loss or maternal outcomes (increased maternal complications, prolonged time to delivery, lower Apgar score) between low transverse cesarean delivery and classic cesarean delivery in the case of extremely premature deliveries <28 weeks [22,24]. In the case of the classic cesarean delivery group, the higher gestation group (above 28 hbd) had an increased risk of maternal complications, such as blood transfusion, endometritis, and ICU admission [24,25].

Complications including hemorrhage (blood loss above 1500 mL, need for blood transfusion, or hysterectomy); infections (endometritis, wound dehiscence, or wound infection requiring antibiotics); reopening; or an unexpected procedure, intensive care unit admission, or death were associated with gestational age, disregarding the method of cesarean section. They were highest between 23 and 27 weeks of gestation [20,25,26].

The indications for fundal uterine incision should be limited to cases such as placenta previa accreta or extremely low-birth-weight infants whose placenta attaches to the whole anterior uterine wall, although it usually seems possible to enter the uterus by performing a J-shaped extension of the low transverse incision [27].

Despite the surgical technique, a cesarean delivery is associated with further long-term complications such as placenta previa, placenta accreta, and cesarean scar pregnancy [16,28].

After the incision of the uterine muscle, the membranes and placenta shall be carefully and manually separated from the uterine wall. The role of the assistant is to ensure a wide enough operating site by pulling the abdominal muscles and angles of the incisions of the uterus and the skin to the sides [7].

**Figure 2:**
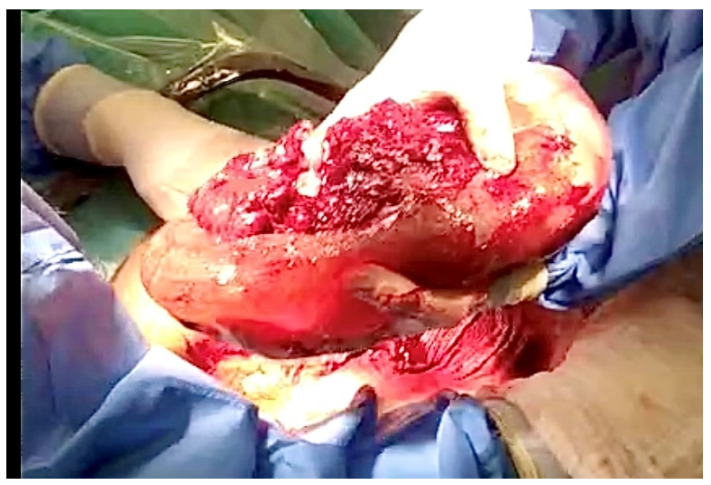
The infant is born within the amniotic sac, which protects the fragile body from the pressure of the uterus and surgeon’s hands. (The picture was taken in the Department of Gynecology, Obstetrics and Neonatology, University Clinical Center in Gdańsk.)

**Figure 3:**
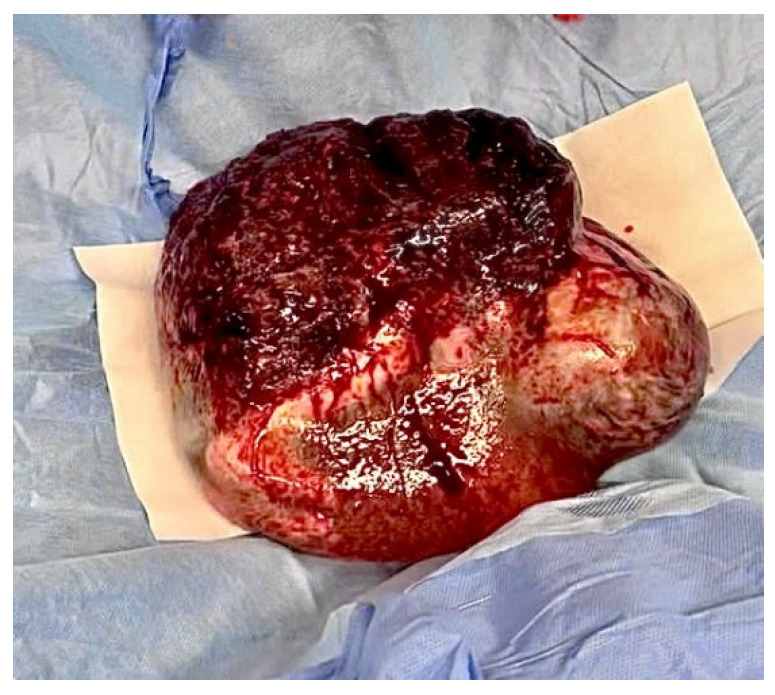
The unit consisting of the neonate, amniotic fluid, amniotic membranes, and placenta is passed to the neonatological team. (The picture was taken in the Department of Gynecology, Obstetrics and Neonatology, University Clinical Center in Gdańsk.)

The fetus should be delivered gently by uterine contractions or gentle pressure applied to the fundus of the uterus in order to stabilize the fetus in a longitudinal lie. The infant is born within the amniotic sac, which protects the fragile body of the newborn from the pressure of the uterus and surgeon’s hands and possible traumas, and passed to the neonatological team together with the placenta [5,7,11] (Figure 2 and Figure 3).

Neonatologists perform the rupturing of membranes at the infant warmer and immediately start necessary stabilization or resuscitation. The time from the separation of the placenta till the onset of resuscitation should not exceed 30 s. In cases of anemia or hypovolemia of the neonate, an own blood transfusion through the umbilical cord may be needed. According to European Resuscitation Council guidelines, deferring cord clamping (DCC) may allow for placental transfusion. It reduces in-hospital mortality and combined death of major disability at 2 years [29,30]. The authors of this review recommend holding the placenta above the preterm neonate during the initial stabilization in the delivery room followed by its clamping after the lung of a neonate has been aerated. Alternatively, umbilical cord milking has been recommended; however, this procedure is contraindicated in preterm infants born below 28th week of gestation due to an increased risk of intracranial hemorrhage [31]. There are also cases describing the amniotic membranes being broken on the mother’s thighs and the neonate being passed to the neonatological team after clamping the umbilical cord, for further care and resuscitation [6,13].

Recently, there have been several trials comparing standard management of the umbilical cord followed by resuscitation, with intact cord resuscitation [32,33,34]. Up to date, there is no strong evidence of better immediate outcomes (oxygen saturation at 5 min) supporting intact cord resuscitation. On the other hand, some data show that intact cord resuscitation (ICR) may lead to a lower number of blood transfusions and milder anemia. Therefore, the last statement of the International Liaison Committee on Resuscitation does not support the routine use of ICR [35].

Murakoshi distinguishes a total and partial “en caul” delivery. Total “en caul” cesarean section means delivering the fetus encased in amniotic fluid and membranes together with the placenta. Blagaić et al. refer to a successful “en caul” cesarean delivery if at least one third of the newborn was delivered in unruptured membranes. They assume that even if the membranes break during the delivery of the second half of neonate’s body, the newborn can still benefit from the protective role of the amniotic fluid and membranes, as the both fundal pressure as well as the pressure of the amniotic fluid wave make it much easier and less traumatic to deliver a whole neonate [5]. This whole compartment is gently handled to the neonatologists for further care. This procedure is recommended for fetuses with an estimated body weight under 1000 g. The partial “en caul” delivery is a case when the membranes are ruptured during the operation and may be used when the estimated body weight is under 1500 g. The “en caul” delivery may also be applied in cases of twin pregnancies or premature rupture of membranes, but the technical challenge is even greater [7].

The work by Lin describes a Pfannenstiel or vertical skin incision and a wide transverse lower segment uterine incision avoiding incising the amniotic membranes. It has been reported that none of the neonates had bruises or fractures after en caul cesarean delivery [1,5]. Abouzeid describes a generous J-shaped lower segment or classical uterine incision [6].

As the last step of the operation, the uterine and abdominal walls are repaired and closed in a typical manner.

## 5. Risk of Exsanguinating and Anemia

Performing an “en caul” cesarean delivery is correlated with the risk of exsanguinating the newborn if the vessels are breached during the separation of the placenta. The plane of the separation is intended as deep as possible to the basal plate by gentle hand movements of the surgeon [1,5,6]. Murakoshi and Jin report that in the majority of cases the placenta separates spontaneously without any help needed or complications [7,13]. The placenta is usually separated in the deep plane of the basal plate and even if the maternal vessels are destroyed during this procedure, it does not affect fetal circulation [1,6]. The potential danger of exsanguinating the newborn can be reduced by squeezing umbilical cord blood towards the fetus before cord clamping, the so-called “milking” movement to ensure better blood supply [1,5]. Abouzaid stated that 12% (3/24) of babies suffered from severe anemia with a first hemoglobin level below 15 mg%. Almost half (11/24) of the studied newborns required blood transfusion. In those cases of neonatal anemia, no other cause was detected (e.g., vasa previa, abruption of placenta, feto-maternal hemorrhage). Therefore, the risk of hemorrhage may be significant [6] (Table 1). On the other hand, works by Shan, Blagaić, and Radzinsky proved that the hemoglobin level did not differ between the groups delivered by “en caul” and conventional cesarean sections [5,11,36].

It is reported that maternal blood loss is not higher during this technique, as the preserved amniotic sac maintains pressure on the uterine incision [5].

Lin et al. describe similar results. In total, 17% (4/23) of neonates had anemia with a hemoglobin level below 14.5 mg%, and 11/24 babies (45%) needed blood transfusion. The average value of the hemoglobin level was 16.1 g/dL. Nevertheless, she assumes that the reasons for blood transfusions were iatrogenic and resulted from frequent blood examinations, essential in preterm newborns and the small overall blood volume of these babies [1]. Those cases show that the pathogenesis of neonatal anemia still remains uncertain and cannot be linked straight with the “en caul” cesarean delivery [14]. Shibata et al. present a case report clarifying a possible reason for the low hemoglobin level (6.7 g/dL) of the neonate—a velamentous insertion of the umbilical cord and dissected blood vessels connecting the fetus with the placenta during the delivery. The membranes enclosing the fetus were completely separated from the surface of the placenta and born as two separate compartments. The blood vessels were most probably broken when the membranes detached from the placenta. After the delivery, blood was still seen dripping from the torn vessels of the membranes. The newborn was heavily anemic and required blood transfusion [14]. The authors suggest that the earlier breaking of the membranes and immediate cord clamping in the operative field could have improved the neonatal results in such a case [14]. In Table 1, a comparison of results of the studies mentioned in this article is presented.

The group of Lin encountered a case of placenta previa and accreta, needing special surveillance. The chosen incision of the uterus was a classical method to spare the placental site. The amniotic sac was delivered with the placenta in situ [1].

The retrospective study performed at a university-affiliated hospital in China by Zhen Jin was performed on a group of 141 successful preterm “en caul” cesarean labors (<37 weeks of gestation) to investigate risk factors for the unsuccessful “en caul” cesarean sections. The uterus was opened performing a lower transverse incision and the amniotic sac was bluntly separated from the uterine walls by the operator, leaving the placental site intact. After delivering the fetus within the amniotic sac, the membranes were ruptured and the newborn was transferred to the neonatologists. Then, the placenta was removed and the uterine and abdominal incisions were closed. The duration of the “en caul” cesarean section was longer than a traditional way of performing cesarean section, resulting from the need to separate the amniotic sac from the uterine wall. Nevertheless, the increase in the risk of hemorrhage was not stated [13] (Table 1).

## 6. Risk of Fetal Asphyxia, Acidemia, and Mortality Rate

The “en caul” cesarean section reduces the risk of fetal asphyxia. The study by the Jin group confirmed that the risk is reduced especially in the group delivered at >34 gestational weeks, although the duration of the procedure was longer than in a conventional method. Preserved membranes and the environment of the amniotic fluid reduce the impact of external stimulation to breathe before delivering the fetal head. Moreover, the fluid prevents the pressure of the uterine muscle and reduces the risk of umbilical cord compression [5,13].

Intrapartum acidemia is a risk factor of respiratory distress and adverse neonatal outcomes. Lin et al. describe levels of arterial pH less than 7.2 in 5 out of 23 babies, and a profound metabolic acidemia less than 7.0 in one case, but those measurements were taken some time after the delivery in the Neonate Intensive Care Unit [1]. Blagaić et al. report no cases of profound metabolic acidemia. In their work, the median value of the first arterial pH measurement was 7.35 ± 0.08 and was higher than in the control group [5] (Table 1).

Jin et al. reported 12 prematurity-related infant deaths, which are 8% of all successfully performed “en caul” cesarean sections. The causes of death were severe asphyxia accompanying intracranial hemorrhage, pulmonary hemorrhage, respiratory distress syndrome, and necrotizing enterocolitis [13]. In the work of Abouzaid, the mortality rate was 29% (26% after excluding one lethal trisomy) [6]. Lin reported a neonatal mortality rate at the level of 4.3% (1/23) [1] (Table 1), whereas Blagaić reported two neonatal deaths unrelated to the method of delivery [5].

## 7. The Optimal Parameters for a Successful en Caul Delivery

In the work by Jin, the failed “en caul” cesarean sections were valued to help optimize the inclusion criteria for this kind of operation [13]. The unsuccessful operation was when the amniotic membranes broke before the complete delivery of the fetus. The average age of pregnancy in this group in the work by Jin was 36 + 1 weeks, and the mean birthweight in this group was 2852 g. The estimated amount of amniotic fluid was approximately 829 mL. Those values are higher than in the group that successfully completed the “en caul” procedure, where the age of gestation was 33 + 2 weeks, mean body weight was 2214 g, and amount of amniotic fluid was 691 mL. This can result from a greater extension of the uterus and the amniotic membranes making them more prone to rupturing [8]. The reason for breaking the membranes lies also in the fact that the structure of amniotic membranes changes near the time of the delivery, making the membranes more elastic, thin, and fragile [41]. Therefore, the highest chance of completing an “en caul” delivery without breaking the amniotic membranes is below 32 weeks of gestation [13]. The work by Jin also revealed that completing the cesarean section successfully in the group below 32 weeks was 92.5%, in the group 32–34 weeks it was 90.6%, and above 34 weeks the success rate was 52.5% [13].

## 8. Indications for a Premature Delivery

It is reported that the leading cause of a premature delivery is preeclampsia (50%). Other reasons include fetal growth restriction (25%) and placental abruption or fetal demise. Indications for a cesarean section which we can encounter in the literature are as follows: the most reported hypertensive disorders including severe preeclampsia and HELLP syndrome, malpresentation correlated with prematurity, and growth restriction [1]. Hypertensive disorders, fetal growth restriction, and fetal distress were the leading indications for a cesarean section in the group investigated by Abouzeid and by Blagaić [5,6]. The study by Radzinsky showed malpresentation and hypertensive disorders as leading indications for the cesarean delivery [11]. Other mentioned indications were placenta abruption and placenta previa with a massive hemorrhage [1] (Table 1).

## 9. En Caul Delivery of Twin Pregnancies

There are also available case reports of an “en caul” delivery of both twins or one of the twins. In the case of twin pregnancies, the mode of the delivery is mostly cesarean section in order to reduce the possible risks of vaginal delivery [36].

The study by Shan et al. analyzes the pregnancy outcomes of preterm (less or equal 32 hbd) twin cesarean deliveries. The evaluated data included the following: duration of the operation, intraoperative hemorrhage, neonatal birth weight, Apgar score, pH level, need for mechanical lung ventilation, length of hospitalization, perinatal neurological damage, intracranial hemorrhage, respiratory distress, need for surfactant infections, and septicemia. The mean operation time was extended in the “en caul” group, but there were no differences in the volume of bleeding nor incidences of postpartum hemorrhage. The extension was correlated by the authors with the time needed for a careful separation of the amniotic membranes. The Apgar score in the first minute of twin A, the Apgar score of 5 and 10 min, pH level and hemoglobin level, hospital stay time, and rate of neurological damage did not vary between the groups, whereas twin B had significantly higher values in the “en caul” group. Lower rates of mechanical ventilation of twin B were observed in the “en caul” group. The respiratory distress rate was lower in both twins, and the rate of intracranial hemorrhage in twin B was higher, but none of these were statistically significant. Furthermore, all the “en caul” patients were divided into two groups on the basis of gestational age and the values were analyzed once more. The “en caul” group above 30 hbd experienced a lower rate of respiratory distress of twin A, and a lower rate of low Apgar scores in the first minute of twin B. In the case of patients below 30 hbd, the rate of respiratory distress was lower whereas the need for mechanical ventilation and the duration of hospitalization did not differ between the “en caul” and control group [36] (Table 1). All those values prove that the “en caul” cesarean delivery is beneficial to both twins. The protective role of amniotic fluid on the fetal head reduces the risk of neurological injuries and umbilical cord compression.

The study by Radzinsky et al. examined 35 twin pregnancies born by transverse “en caul” cesarean section and 35 pairs of twins delivered by the use of a conventional cesarean section, all divided into groups based on gestational age: 28–30 weeks, 31–33 weeks, and 34–37 weeks. The analyzed parameters were as follows: caesarean section duration, Apgar score, need for mechanical lung ventilation and its term, neurological state, length of hospital stay, further physical and neurological development, and morbidity of neonates during the first year of life. The results showed that the neonates born “en caul” were in a better condition (higher Apgar score, less use of mechanical ventilation, fewer cases of periventricular hemorrhage and cerebral ischemia) and further neurological disorder morbidity rates for the first year of life were lower than in the control group because of the protective role of the amniotic fluid and preserved membranes during the delivery. No differences in the hemoglobin level were observed between the groups, and therefore the “en caul” delivery did not elevate the risk of anemia. The authors stated that the delivery time was extended in the study group by 2.5 min in comparison to in the control group [11] (Table 1).

Pal reports a successful “en caul” delivery of both twins at 37 hbd with weights of 2200 g and 1500 g. The operation and the postoperative period were uncomplicated [4].

A case report by Prabaker describes an “en caul” cesarean delivery of the second twin. There was a leakage of amniotic fluid from the sac of the first twin. The delivery occurred at 28 hbd and no complications were described [37].

Zacharis describes a case of a cesarean twin delivery at 38 hbd, where the “en caul” delivery was applied only to the second twin [3].

Also, Sattiraju et al. report an “en caul” delivery of twins by cesarean section [42].

## 10. En Caul Vaginal Deliveries

In the literature, there are many examples of a vaginal delivery “en caul”. The factors predisposing to “en caul” deliveries are low gravida and prematurity. The group of Malik reports a case of a multiparous woman giving birth to a child at 23 weeks of gestation. The newborn required emergent management resulting from extreme prematurity—respiratory distress, impaired thermoregulation, the possibility of sepsis, as well as anemia and intraventricular hemorrhage. Death occurred at 5 days of life [38].

Moran reported a case of a spontaneous vaginal delivery of an unviable fetus in an Emergency Department at the gestational age 19 + 6 weeks [39].

Richmond et al. describe in their work many “en caul” vaginal deliveries. They reported that the mortality rate of infants was similar disregarding the method of delivery, whether cesarean or vaginal [43].

Gosh et al. describe a spontaneous vaginal “en caul” delivery at 32 weeks of gestation induced by the mother’s infection (pyelonephritis) with an Apgar score 4/6/8, born within a small amount of amniotic fluid but without any signs of sepsis. The authors propose oligohydramnios to be one of the reasons for an en caul delivery [12].

Girault et al. studied a group of 123 extremely preterm singletons in breech presentation, at the age of pregnancy 24 to 27 + 6 weeks. In total, 52 were included in the “en caul” group and 71 in the “ruptured membrane” group. The average age of pregnancy at the time of the delivery was 25 + 3 in the “en caul” group and 26 + 0 in the control group. The rate of antenatal corticosteroids administrated, the number of intrauterine infections, and fetal extraction obstacles in both groups were comparable. They stated that the perinatal mortality did not differ between those groups. The deaths of infants from the “en caul” technique occurred rather in the NICU units, whereas the “ruptured membranes” group experienced deaths more often intrapartum or soon after the delivery. In the main analysis, they reported fewer cardiovascular abnormalities in the “en caul” group. The pH cord measurements were better in the “en caul” group. The “en caul” technique did not prove the decrease in perinatal mortality in general. Nevertheless, after the delivery, the “en caul” group had a better neonatal state [40].

An EPIPAGE2 analysis confirmed that a cesarean section before 26 weeks of pregnancy is correlated with a higher risk of maternal mortality and morbidity in comparison to a vaginal delivery. Therefore, it is essential to find the most appropriate way to deliver such small infants and minimize possible consequences to the mother. It seems relevant to take a vaginal “en caul” breech delivery into consideration. This technique can minimize the trauma correlated with maneuvers and traction necessary to deliver the fetus from breech presentation or risk of head entrapment. The amniotic sac can also prevent engagement of the fetus in birth canal while the cervix is not fully dilatated [40].

## 11. Conclusions

Sparse available studies on “en caul” deliveries may lead to the limitation of our work because there are few available studies on the subject resulting from poor popularity and training for obstetricians to perform cesarean section while preserving the amniotic sac. Another limitation might be the fact that the premature rupture of membranes is among the leading causes of premature deliveries and a preserved amniotic membrane is crucial for an “en caul” delivery.

The “en caul” cesarean section is an effective method to deliver preterm newborns unharmed. The mode of delivery of this group of children is still discussed. Nevertheless, it seems that an amnion protective cesarean section would be optimal for preterm fetuses with low birth weight, as a method to prevent neonatal trauma including intracranial hemorrhages.

Preserved amniotic membranes during a delivery protect the fragile body of a newborn, mainly its head, from the pressure trauma, and they reduce fetal distress accompanying a delivery. The manifold benefits include lower danger of asphyxia, a higher cord pH measurement, a higher Apgar score, and a decreased risk of an entrapment by a suddenly contracted uterus.

We still have to bear in mind the future risk for the mother and higher morbidity. A preterm cesarean section is often correlated with a higher incidence of subsequent uterine rupture or impaired placentation in future.

## Figures and Tables

**Table 1:** Characteristics of studies included.

Author	Including Criteria	Study Group	Gestational Age at the Delivery	Birth Weight	Way of the “en caul” Delivery	Rupture of Membranes During the Delivery	Indications for a Cesarean Section	Mortality Rate	Apgar Score (1, 5, 10 min)	pH	Hb [g/dL]
Lin Ch. [1]	EBW < 1500 g or GA ≤ 32 hbd	20 women 23 neonates 16 single pregnancies 4 twin pregnancies 10 breech 1 transverse 12 vertex	28^+0^ ± 4^+5^	1073 ± 300 g 5/23 < 3 pctl	Cesarean section -Pfannenstiel or vertical skin incision -19 lower transverse uterine incisions -1 classical uterine incision (placenta accreta)	3/20 spontaneously during the delivery	-9/20 hypertensive disease -2/20 placenta abruption -2/20 placenta previa with antepartum hemorrhage -2/20 fetal distress -1/20 maternal pulmonary hypertension -22/23 fetuses with growth restriction	4.3% (1/23) Twin A 240 g, admitted with premature rupture of membranes, entrapped in the cervix	15/23 ≥ 7 (5 min) (65.2%)	7.278 ± 0.117 1/23 < 7	16.1 ± 2.1 g/dL
Abouzeid H. [6]	EBW < 1500 g or GA ≤ 32 hbd	24 single pregnancies 11 cephalic 6 breech 7 not recorded	24^+0^ − 32^+0^	410–1480 g	-20 horizontal lower segment uterine incisions -1 classical incision -J-extension of the lower segment incision -2 unspecified	5/24 during the delivery 7/24 unclear description	-14/24 hypertensive disease -10/24 fetal distress/growth restriction	29% (7/24) 1 trisomy 13			3/24 below 15
Jin Z. [13]		211 singletons’ cephalic presentation	28^+0^ − 36^+9^	940–3560 g		70/211 (66.8%)		12 Intracranial hemorrhage, pulmonary hemorrhage, neonatal RDS, NEC			
Blagaić V. [5]	26–35 hbd and EBW 700–1500 g	10 singleton pregnancies	28^+1^ − 34^+6^	1140 g (700–1370 g)	Lower segment cesarean section	0/10	IUGR, oligohydramnio, HELLP, fetal distress, preeclampsia	2/10	9, 9, -	7.32–7.43	13.4–24.6 g/dL
Shibata T. [14]		1 singleton pregnancy	26		Lower segment cesarean section						6.7 g/dL
Shan D. [36]	GA ≤ 32 hbd Twin pregnancy	90 “en caul”		1400.72 ± 280.6 1347.82 ± 286.4	Lower segment cesarean section	0/90				7.33 ± 0.10 7.32 ± 0.09	16.4 g/dL 16.8 g/dL
Radzinsky W. [11]	GA 28–37 hbd Both fetuses alive No PROM in at least one twin	35 twin pregnancies	28–37 weeks (28–30) (31–33) (34–37)		Lower segment cesarean section	0	Breech presentation of twin A, shoulder presentation, preeclampsia, hypoxia, prolonged labor, uterine scar, IVF procedure, PROM of one twin	0	Twin A/B at 5 min: (GA 28–30) 6/6 (GA 31–33) 7/7 (GA 34–37) 8/7.5		Similar
Pal A. [4]		1 twin pregnancy Twin A—footling presentation	37	2200 g; 1500 g	Lower segment cesarean section	0	Severe pregnancy-induced hypertension	0	4, 6, 8; 4, 6, 8.		
Prabaker Ch. [37]		1 twin pregnancy Twin A—cephalic Twin B—breech	28		Cesarean section	Twin A					
Malik R. [38]		1 singleton	23	497 g	Vaginal delivery	0		100% At the 5th day, grade 3 intraventricular hemorrhage bilaterally and an early grade 4 bleeding in the left cerebral hemisphere	4, 6, 8		
Moran M. [39]		1 singleton unviable	19 + 6		Vaginal delivery			Intrauterine demise			
Girault A. [40]		52 singletons in breech presentation	24 − 27^+6^	783 ± 125 g	Vaginal delivery			19.2%	5.5 ± 2.7 (5 min)	7.32 ± 0.1	

GA—gestational age; EBW—estimated body weight; RDS—respiratory distress syndrome; NEC—necrotizing enterocolitis; IUGR—intrauterine growth restriction; HELLP—hemolysis, elevated liver enzymes, low platelets; IVF—in vitro fertilization; PROM—premature rupture of membranes.
